# Association of Coronary Computed Tomography Angiography and Stress Echocardiography with Long-Term Cardiac Outcome: A Comparison Study

**DOI:** 10.3390/jcm12030903

**Published:** 2023-01-23

**Authors:** Nicola Gaibazzi, Fausto Rigo, Valentina Lorenzoni, Cristina Pasqualetto, Alberto Foà, Enrico Cagliari, Nicola Cavasin, Andrea Botti, Chiara Martini, Domenico Tuttolomondo

**Affiliations:** 1 Department of Cardiology, Parma University Hospital, 43126 Parma, Italy; 2 Division of Cardiology, Villa Salus Hospital Foundation/IRCCS San Camillo, 30126 Venice, Italy; 3 Istituto Sant’Anna, 56127 Pisa, Italy; 4 Department of Cardiology, Ospedale Civile di Dolo, ULSS 3 Serenissima, 30174 Venice, Italy; 5 Unit of Cardiology, Department of Experimental, Diagnostic and Specialty Medicine—DIMES, University of Bologna, 40126 Bologna, Italy; 6 Neuroradiology Department, Mestre Hospital, ULSS3 Serenissima, 30174 Mestre, Italy; 7 Department Diagnostic, Parma University Hospital, 43126 Parma, Italy; 8 Department of Medicine and Surgery, University of Parma, 43121 Parma, Italy

**Keywords:** stress echocardiography, coronary computed tomography angiography, coronary artery calcium score, Doppler coronary flow velocity reserve, chronic coronary syndrome, long-term cardiac outcome

## Abstract

Aims: This study aimed to assess which variables on coronary computed tomography angiography (CTA) and vasodilator stress-echocardiography (SE) are best associated with long-term cardiac outcome in patients presenting for suspected chronic coronary syndrome (CCS) who performed both tests. Methods: We identified 397 patients with suspected CCS who, between 2007 and 2019, underwent both SE and CTA within 30 days. Coronary artery calcium score (CACS) and the number of coronary arteries with diameter stenosis >50% were assessed on CTA. The presence of reversible regional wall motion abnormalities (RWMA) and reduced Doppler coronary flow velocity reserve in the left-anterior descending coronary artery (CFVR) were assessed on SE. The association of SE and CTA variables with cardiac outcome (cardiac death or myocardial infarction) was evaluated using Fine and Gray competing risk models. Results: During a median follow-up of 10 years, 38 (9.6%) patients experienced a nonfatal myocardial infarction and 19 (4.8%) died from a cardiac cause. RWMA (HR 7.189, *p* < 0.001) and a lower CFVR (HR 0.034, *p* < 0.001) on SE, along with CACS (HR 1.004, *p* < 0.001) and the number of >50% stenosed coronary vessels (HR 1.975, *p* < 0.001) on CTA, were each associated with cardiac events. After adjusting for covariates, only CACS and CFVR remained associated (both *p* < 0.001) with cardiac outcome. Conclusion: Our data suggest that only CFVR on vasodilatory SE and CACS on CTA are independently and strongly associated with long-term cardiac outcome, unlike RWMA or the number of stenosed coronary arteries, usually considered the hallmarks of coronary artery disease on each test.

## 1. Introduction

In the challenging clinical scenario of suspected chronic coronary syndrome (CCS), few studies have evaluated the prognostic role of coronary computed tomography angiography (CTA) versus functional imaging, and none have specifically compared the long-term prognostic value of CTA with stress echocardiography (SE) [1,2,3]. Furthermore, no data are currently available regarding the association of such diagnostic tests with outcome when both tests were performed in the same cohort of patients. Within SE assessment, the availability of the Doppler coronary flow velocity reserve measurement in the left anterior descending coronary artery (CFVR) has been proven to increase the prognostic yield because it has been identified as the single most accurate variable for risk stratification on SE, available only from vasodilator SE [4,5,6,7]. It is worth mentioning that two previous studies tried to compare CTA and SE with CFVR, but the short follow-up time or paucity of events recorded strongly limited the results [8,9].

To fill this gap, we selected all patients with suspected CCS who underwent both CTA and SE within a short time lapse, and we evaluated the long-term combined endpoint of nonfatal myocardial infarction or cardiac death. The association of CTA and SE variables with the combined endpoint was assessed, aiming to evaluate coronary artery calcium score (CACS) and the number of stenotic vessels (stenoses > 50%) on CTA, as well as the presence of reversible wall motion abnormalities (RWMA) and reduced CFVR (stress/rest ratio < 2) on SE, after adjusting for all available clinical and imaging covariates.

## 2. Materials and Methods

### 2.1. Patients

From the SE database of Venice hospitals (Italy) we selected all patients with suspected CCS who, in the period between 2007 and 2019, underwent both SE with CFVR measurement available and CTA within 30 days, regardless of which test was performed first. If coronary revascularization or any cardiac event occurred during the short time between the two tests, the patient was excluded from the study. Diabetes mellitus was defined as a fasting plasma glucose level ≥ 126 mg/dL, hemoglobin A1c ≥ 6.5% (48 mmol/mol), or the need for insulin or oral hypoglycemic agents. Hypercholesterolemia was defined as total cholesterol ≥ 200 mg/dL (5.2 mmol/L) or treatment with lipid-lowering medications. Hypertriglyceridemia was defined as fasting triglycerides ≥ 150 mg/dL (1.7 mmol/L). Hypertension was defined as blood pressure > 140/90 mmHg or use of antihypertensive medication.

### 2.2. Stress Echocardiography

Philips ie33 (Philips Medical Systems) was used with a standard S5 transducer. Our protocol for accelerated high-dose dipyridamole SE was used. Briefly, it consists of rest and peak vasodilation assessments of the following imaging parameters: regional wall motion and spectral Doppler CFVR for peak diastolic velocity stress/rest ratio in the mid-distal left anterior descending artery. Stress acquisition was performed after dipyridamole administration of 0.84 mg/kg infused in 6 min; RWMA and CFVR were the only mandatory assessments during the protocol [10]. The left ventricle was divided into 17 segments according to the recommendations of the American and European Societies of Echocardiography [11]. Segmental wall motion was graded as follows: normal = 1; hypokinetic = 2; akinetic = 3; dyskinetic = 4. Reversible ischemia was defined as the occurrence of a stress-induced new dyssynergy or worsening of rest hypokinesia in ≥1 segment. CFVR < 2 was considered abnormal (also termed “reduced”).

### 2.3. Coronary Computed Tomography Angiography

Most coronary CTA examinations were performed using a Dual-Source CT system (Somatom Definition FLASH, Siemens Healthcare, Forchheim, Germany), although a minority of patients (31, years 2007–2009) were imaged with a 64-slice scanner (Siemens Somatom). The dataset was analyzed by two experienced readers using an offline workstation software package (Leonardo, Siemens Medical Solutions, Forchheim, Germany). CTA image acquisition was performed according to the current practice guidelines [12]. Angiographic datasets of the reconstructed coronary vessels were created in the best phase of the cardiac cycle depending on the heart rate of the patient. All coronary segments were analyzed in accordance with the American Heart Association classification [13]. The presence of atherosclerotic plaques was assessed using axial images, multiplanar reconstruction, and cross-sectional reconstruction. For the final grading of coronary artery disease (CAD) and stenosis severity in this study, we used the following criteria for each coronary artery: (I) no visible plaque or presence of at least one plaque with nonobstructive stenosis (maximal diameter stenosis ≤ 50%); (II) presence of potentially obstructive stenosis (maximal diameter stenosis > 50%). Left main trunk with a stenosis > 50% counted as two diseased vessels, and, if at least one of its main branches (left anterior descending or circumflex or intermediate coronary artery) also showed a stenosis > 50%, this resulted in an overall count of three vessels with a stenosis > 50%; in this case, if the right coronary artery was also affected by a stenosis > 50%, the number of diseased vessels consequently could reach a maximum score of four diseased vessels. Therefore, the number of diseased vessels was potentially in the 0–4 range. Coronary calcium was measured in non-contrast electrocardiography-triggered CTA. Coronary artery calcium score was classified using a threshold of 130 Hounsfield units (HU) involving more than three contiguous voxels for identification of a calcific lesion resulting in a minimum lesion area of 1.02 mm^2^. The lesion score was calculated using the area density method, by multiplying the lesion area by a density factor derived from the maximal HU within the area, as described by Agatston [14]. CACS was calculated with commercially available software (CaScore; Siemens, Germany).

In the classification of calcified atherosclerotic burden, we defined the following commonly used groups: CACS = 0, CACS = 1 to 99, CACS = 100 to 399, and CACS >399) regardless of whether patients had nonobstructive or obstructive CAD [15,16].

### 2.4. Follow-Up

Outcome was determined from patients’ interview at the outpatient clinic, hospital chart reviews, and telephone interviews with patients, close relatives, or referring physicians. Cardiac death and nonfatal myocardial infarction were registered as cardiac events; death from other causes was also collected and considered in the analysis as a competing event. Coronary revascularization (surgical or percutaneous) was also recorded. Myocardial infarction was defined by cardiac enzyme changes in association with at least one of the following criteria: (I) symptoms of acute myocardial ischemia; (II) new ischemic electrocardiogram changes or development of pathological Q waves; (III) imaging evidence of new loss of viable myocardium or new regional wall motion abnormality; (IV) identification of a coronary thrombus by coronary angiography according to fourth universal definition of myocardial infarction [17]. Cause of death was based on medical records and death certificates. The diagnosis of cardiac death required documented life-threatening arrhythmias, cardiac arrest, death attributable to congestive heart failure, or myocardial infarction in the absence of any other precipitating factor. Sudden unexpected death was classified as a cardiac death when an obvious noncardiac explanation was excluded.

Follow-up data were analyzed for the association with cardiac events, and only the first documented event was considered, terminating the follow-up per protocol (censoring).

### 2.5. Statistical Analysis

Categorical variables were presented as numbers and relative frequencies (percentages); continuous variables were presented as the mean ± SD or median with interquartile range (IQR), according to their distribution. Crude event rates were used to describe the risk of cardiac events according to combination of imaging variables. Considering cardiac events as the main outcome variable and death from other causes as competing event, the Fine and Gray competing risk model was used to characterize cumulative incidence functions for the main outcome variable on the basis of each of the four parameters assessed: CACS data and number of vessels with >50% stenosis (on CTA); RWMA and CFVR (on SE). Using the Fine and Gray regression analysis, we also evaluated independent predictors of the subdistribution hazard ratios in the presence of competing risks. Variables associated with the risk of cardiac events were then evaluated considering Fine and Gray competing risk regression models, and results were expressed as the hazard ratio (HR) and 95% confidence interval (CI). Clinical predictors considered in the competing risk models were defined according to the Framingham risk score assessment and included age, gender, obesity, diabetes, hypercholesterolemia, hypertension, and family history of premature CAD (before 55 years for men or 65 years for women); revascularization and/or the presence of known angiographic CAD prior to the tests were also considered risk factors [18]. Coronary revascularization procedures performed after SE and CTA were also considered among the covariates. Rest left-ventricle ejection fraction (LVEF) was analyzed both as a continuous variable and as a dichotomous variable (>50% or <50%); SE parameters considered were the presence of RWMA and CFVR <2 versus >2, while CTA parameters were CACS and the number of coronary vessels with at least one stenosis >50% (on a 0–4 number of vessels, as described above).

All variables with *p* < 0.1 at univariable analysis were considered for the inclusion into multivariable models. Proportional hazards assumption was evaluated using visual inspection of the log–log survival curves and the Schoenfeld residuals test. Time-dependent ROC curves and estimated areas under the curve (AUCs) were calculated to assess the accuracy of variables identified in the multivariate analysis not including and including CACS or CVFR.

A *p*-value <0.05 was considered statistically significant. STATA release 15 and R version 3.6.2 were used for analysis. The study was approved by the local ethics committee (183A/CESC) and was performed in accordance with the ethical standards of the Declaration of Helsinki and its later amendments.

## 3. Results

### 3.1. Outcome

Three hundred ninety-seven patients satisfying the inclusion criteria were identified and enrolled. During a median follow-up of about 10 years (median 3783 days, lower–upper quartile, 1470–4653), 38 patients (10%) experienced a nonfatal myocardial infarction, 19 (5%) died because of cardiac causes, and 26 (7%) died from causes other than cardiac. Four patients had first a nonfatal myocardial infarction, and later died because of cardiac causes during follow-up, such that the final number of cardiac events was 53. According to caring physicians’ judgement, 54 (14%) subjects underwent coronary revascularization (11 surgical and 43 percutaneous revascularization) during the follow-up period, at a median of 759 (lower-upper quartile, 131–1153) days after the last of the two performed tests.

### 3.2. Study Population

Table 1 shows the full demographic data, baseline clinical risk factors, medications, and LVEF, together with SE and CTA data. In the study group, the median age was 65 years (SD = 11); males represented approximately two thirds (69%) of the study cohort, and mean LVEF was 56% (SD = 8).

### 3.3. Imaging Data

Stress echocardiograms were positive for reversible ischemia according to the presence of RWMA in 73 (18%) patients, while CFVR was reduced (<2) in 121 (31%) patients. CFVR median value in the study group was 2.2 (SD = 0.5). The median value of CACS was 47 Agatston units (lower–upper quartile 6–245), and 176 (44%) patients had at least one coronary artery affected by CAD with at least one >50% stenosis.

**Figure 1:**
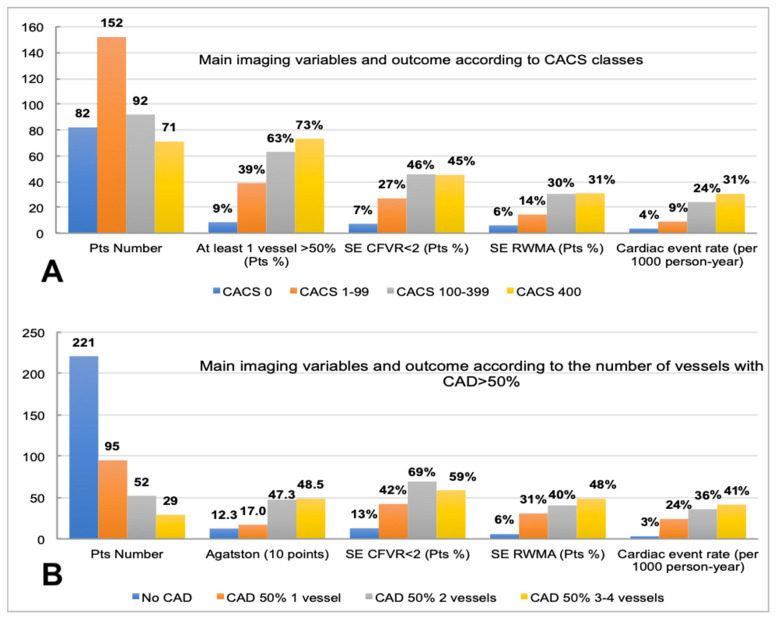
Relationship of CACS categories (**A**) and number of vessels with CAD >50% (**B**) with the other imaging variables and cardiac event-rate. CACS, coronary artery calcium score; CAD, coronary artery disease; SE, stress echocardiography; CFVR, coronary flow velocity reserve; RWMA, reversible wall motion abnormalities. Median Agatston score is divided by 10 to fit the graph vertical scale. Crude cardiac event rate expressed in terms of events per 1000 person-years is reported.

Figure 1 shows the multiple interrelations between either CACS categories (Figure 1A) or the number of stenosed coronary arteries >50% (Figure 1B), with the distribution of the other imaging variables collected and the corresponding event rate.

Coronary artery calcium score classes were associated with the number of >50% stenosed vessels, and the outcome associated with such classifiers was distributed across a wide range of event rates, either when using CACS classes (from 4 per 1000 person-years for CACS = 0 to 31 per 1000 person-years for CACS > 399) or the number of diseased coronary vessels (from 3 per 1000 person-years for no vessel with CAD > 50% to 41 per 1000 person-years for three or four vessels with CAD > 50%).

Figure 1 highlights that a CACS finding beyond the third class (100–399) was not associated with a progressively higher percentage of patients with positive SE (either according to RWMA or reduced CFVR) (Figure 1A), while, when the number of vessels with at least one stenosis > 50% was considered (Figure 1B), the percentage of patients with RWMA kept progressively increasing across the four categories; this was not the case for the percentage of patients showing reduced CFVR. While the most represented class of CACS in the study population was 1–99, the most numerous subgroup of patients according to the number of vessels with CAD > 50% was the category with no stenosed vessels; this highlights that, as expected, there was a relevant percentage of patients without potentially obstructive CAD who still had detectable coronary calcium [19].

### 3.4. Cumulative Incidence Function

**Figure 2:**
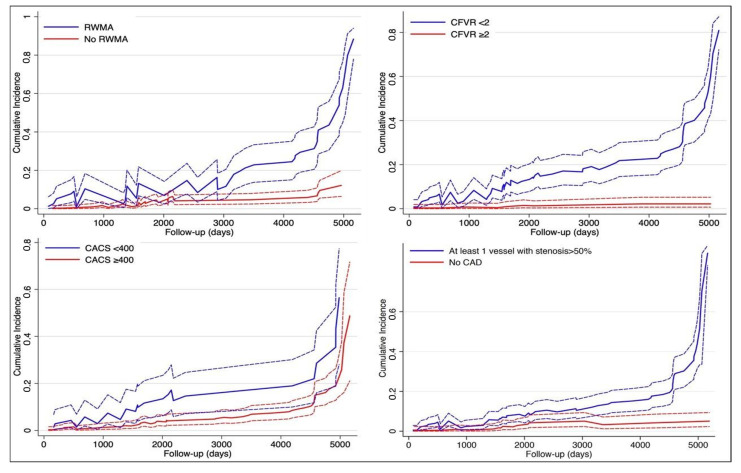
Cumulative incidence of cardiac events along with 95% confidence bands comparing the presence of RWMA, CFVR < 2, CACS ≥ 400, and number coronary arteries with stenosis > 50%, accounting for competing events. Abbreviations are as in Figure 1.

Figure 2 shows the cumulative incidence of cardiac events along with 95% confidence bands, based on the presence of RWMA (Figure 2, upper left panel), normal or reduced CFVR (Figure 2 upper right panel), or more than moderate (CACS > 399) coronary calcification or number of stenosed coronary arteries (Figure 2, lower panel). The cumulative incidence was only slightly higher in presence of RWMA, CACS > 399, or stenosed coronary arteries, but was instead markedly higher for patients with reduced CFVR, as visually evident.

### 3.5. Association of Clinical and Imaging Variables with Cardiac Events

Table 2 (left column) shows the data regarding the univariate association of clinical and imaging variables with cardiac events considering univariable competing risk models. LVEF (HR 0.954, 95% CI: 0.921 to 0.987, *p* = 0.007) and having undergone elective coronary revascularization after the tests (HR 2.629, 95% CI: 1.525–4.531, *p* = 0.001) were significantly associated with outcome on univariate analysis, while known CAD showed *p* < 0.1 (HR 1.690, 95% CI: 0.946–3.019, *p* = 0.077) and was, thus, considered as candidate variable on multivariable analysis.

Each of the four imaging variables assessed in the current study (two assessed using SE and two assessed using CTA) was also significantly associated with outcome at univariable analysis. Specifically, the presence of ischemia in the form of RWMA during SE was associated with cardiac events (HR 7.189, 95% CI: 4.112 to 12.569, *p* < 0.001), as well as for a lower CFVR (HR 0.034, 95% CI: 0.017 to 0.067, *p* < 0.001), higher CACS (HR 1.004, 95% CI: 1.002–1.006, *p* < 0.001 for each 10-point increase), and the number of diseased coronary vessels (with >50% stenosis) diagnosed on CTA (HR 1.975, 95% CI: 1.591 to 2.453, *p* < 0.001).

### 3.6. Multivariable Competing Risk Model (as Shown in Figure 3)

Table 2, in the mid and the right columns, shows the results from two different multivariable models adapted according to the results from the univariable analysis: a first model in which LVEF, CFVR, and CACS were considered as continuous variables, and the other considering such variables categorized according to clinically relevant cutoffs. In the multivariable competing risk model including continuous parameters, among the variables potentially associated with outcome (*p* < 0.1 at univariable analysis) only two imaging variables remained significantly associated with cardiac events: CACS (HR 1.004, 95% CI: 1.002 to 1.006, *p* = 0.001 for each 10-point increase) and CFVR (HR 0.044, 95% CI: 0.014 to 0.142, *p* < 0.001). When considering CVFR as a categorical parameter (<2) and CACS in the usual four categories, only CFVR < 2 remained significantly and independently associated with the risk of cardiac events (HR 13.890, 95% CI: 4.154 to 46.445, *p* < 0.001).

## 4. Discussion

Patients with suspected CCS represent an abundant and heterogeneous clinical entity with still unresolved scientific evidence, especially regarding the diagnostic approach. For instance, in the functional imaging arm of the PROMISE study, SE was one of the allowed modalities to be compared with the CTA arm, but only 20 events were recorded in the SE subgroup, thus impairing a thorough evaluation of SE versus CTA. Conversely, in the other existing randomized study comparing functional imaging versus CTA, only 30 patients underwent SE [1,2]. As a result, there is currently no solid evidence on which tests between CTA or SE (with additional CFVR measurement) better correlate with long-term cardiac outcome in patients with suspected CCS.

In our retrospective study, a cohort of patients with suspected CCS following a clinical indication underwent both SE and CTA within a short time lapse, and they were followed for more than 10 years to evaluate the long-term cardiac outcome.

It came with no surprise that the two variables collected during SE, as well as both variables collected from CTA, were all individually associated with the long-term outcome of cardiac death or nonfatal myocardial infarction, but only CFVR on SE and CACS (although only as a continuous variable) on CTA remained significantly and strongly associated with cardiac events in the multivariable assessment. In fact, after adjusting for the other clinical and imaging variables, the presence of RWMA and the number of >50% stenosed coronary vessels were not significantly associated with the long-term outcome. Notably, age and elective revascularization during follow-up were the only two clinical variables associated with prognosis in multivariable competing risk models.

Coronary artery calcium score and CFVR, although they originate from two different types of tests (anatomical or functional), share a higher sensitivity for milder or early-stage atherosclerotic disease when compared with their respective traditional, theoretically “ischemia hallmark” counterparts (obstructive CAD on CTA and reversible RWMA on SE).

We speculate that the tighter association of both CACS and CFVR with outcome in this study confirms the capability of these two markers to detect those early and possibly preclinical signs of CAD, which may turn into clinical events in the very long run.

A paradigm change in our understanding and management of CAD has recently taken place, after the ISCHEMIA trial results indirectly suggested (and later prospective CTA studies proved) that the overall plaque burden of CAD is in fact more strictly associated with prognosis rather than the severity of single coronary lesions, e.g., defined in terms of percentage luminal stenosis [19,20,21,22]. Therefore, it was not surprising that CACS, a robust surrogate of the overall atherosclerotic burden in the coronary arteries, in our study outperformed an index of apparently more severe (but focal) CAD, represented by the number of coronary vessels with at least one luminal stenosis >50%.

As far as SE is concerned, diffused but not yet obstructive CAD may be able to impair a functional marker such as CFVR, often in the absence of reversible RWMA, which is generally found only in presence of severe “critical” coronary blood flow reductions, mostly subtended by severe coronary artery stenosis.

Therefore, RWMA during SE (not unlike all other types of stress tests relying on wall motion assessment), with its binary “normal/abnormal” segmental result, is not expected to be associated with outcome as strictly and proportionally as CFVR does. The functional CFVR measurement is potentially capable of addressing the continuous spectrum of coronary flow reserve reduction, inversely proportional to the overall burden of coronary artery disease and influenced by microvascular function. Additionally, as recently demonstrated by our group, a reduced CFVR may underlie a low-grade coronary inflammation, which supposedly represents one of the earliest steps in atherosclerosis [23].

Overall, the CFVR measurement enhances the diagnostic performance of SE, limiting the existing gap with CTA which is clearly able to detect a CAD via CACS assessment that, albeit not necessarily related to ischemia, still represents a high risk marker.

## 5. Limitation

The single-center retrospective nature of our study surely represents a limitation, and the results should be interpreted with caution. Not unlike other retrospective studies, there was potential bias in the selection of the study population; it was per-protocol required that both SE and CTA, whichever first, were performed on clinical grounds, potentially selecting a special case mix of patients requiring a second test (either one) to reach a final diagnosis. In the case of patients first undergoing SE, the typical reason to indicate CTA as a second test was a borderline SE result (either for borderline RWMA findings or unexpectedly low CFVR in presence of normal wall motion behavior). In patients undergoing CTA first, a functional test was generally required either when an intermediate coronary stenosis was detected, or to establish the presence of reversible ischemia in case of a clearly obstructive stenosis; patients without significant CAD on CTA usually did not undergo SE. In the current study case mix, there was a prevalence of the first scenario, since 83% of patients underwent SE first while CTA was the first test in the remaining 17%. Our results may not apply to dobutamine or exercise SE or other imaging modalities, where ischemia is often considered more likely to induce RWMA. Generalizability of the study conclusions to a general population of patients with suspected CCS may be limited and require larger studies for confirmation.

## 6. Conclusions

SE and CTA results were associated with long-term outcome of cardiac death or nonfatal myocardial infarction in patients with suspected CCS. After the adjustment for covariates, CFVR and CACS outperformed—and obscured—the main hallmarks of CAD available on each test, i.e., RWMA on SE and the number of stenosed vessels on CTA, which were not significantly associated with outcome in the multivariable competing risk model. The current study suggests that CFVR and CACS should be the variables primarily considered on SE and CTA in the long-term cardiac risk assessment of patients with suspected CCS.

## Figures and Tables

**Figure 3:**
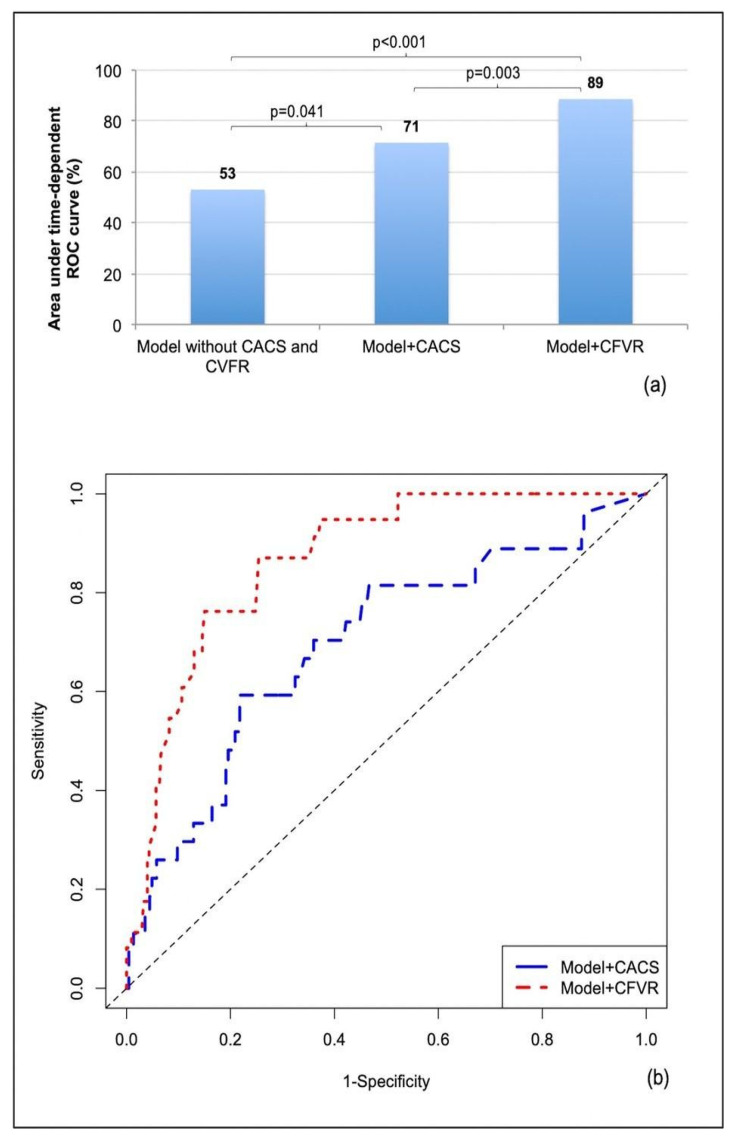
Accuracy of different models including clinical and imaging variables (not comprising CACS and CFVR and comprising either CACS or CFVR) for the prediction of risk of cardiac events, accounting for competing events. (**a**) Area under time-dependent ROC curves (AUC) as a percentage for the reference model including LVEF, RWMA, elective revascularization and number of vessels with coronary stenosis ≥ 50%, and the same model with the addition of either CACS or CFVR, as well as pairwise comparison for the improvement of AUC with the addition of CACS or CFVR. (**b**) Time-dependent ROC curves for the models including clinical and imaging variables, with the addition of either CACS or CFVR. Abbreviations are as in Figure 1. ROC, receiver operating curve.

**Table 1:** Baseline clinical characteristics, coronary computed tomography angiography and vasodilator stress-echocardiography findings in the study patients.

Study Patients (n = 397)	
**Age in years, mean ± SD**	65 ± 11
Male gender, n (%)	272 (68.5)
**Risk factors**	
Hypertension, n (%)	236 (59.4)
Hypercholesterolemia, n (%)	215 (54.2)
Smoking, n (%)	124 (31.2)
Diabetes, n (%)	75 (18.9)
Family history of CAD, n (%)	132 (33.2)
**Medical history**	
Known CAD (MI or/and elective revascularization), n (%)	10 (2.5)
-Previous PCI, n (%)	4 (1)
-Previous CABG, n (%)	4 (1.)
**Medications at the time of stress echocardiography**	
Statins, n (%)	159 (40.1)
Beta-blockers, n (%)	195 (49.1)
Aspirin, n (%)	163 (41.1)
**Echocardiography**	
LVEF, mean ± SD	56 ± 8
LVEF reduced (<50%), n (%)	51 (12.9)
Rest RWMA, n (%)	73 (18.4)
**Stress echocardiography**	
Inducible RWMA, n (%)	77 (19.4)
Delta wall motion score index, median [min; max]	0.05 [0; 0.56]
CFVR < 2, n (%)	121 (30.5)
**Coronary computed tomography angiography**	
At least one vessel with coronary stenosis ≥ 50%, n (%)	176 (44.3)
One vessel with coronary stenosis ≥ 50%, n (%)	95 (23.9)
^1^ Two vessels with coronary stenosis ≥ 50%, n (%)	52 (13.1)
^1^ Three vessel with coronary stenosis ≥ 50%, n (%)	24 (6.1)
^1^ Four vessel with coronary stenosis ≥ 50%, n (%)	5 (1.3)
CACS = 0, n (%)	82 (20.7)
CACS < 99, n (%)	152 (38.3)
CACS = 100–399, n (%)	92 (23.2)
CACS ≥ 400, n (%)	72 (18.2)
**Outcome/ Follow-up**	
Nonfatal MI	38 (9.6)
Cardiac death	19 (4.8)
Death from other causes	26 (6.5)
PCI during follow-up, n (%)	43 (10.8)
CABG during follow-up, n (%)	11 (2.8)

CAD, coronary artery disease; MI, myocardial infarction; PCI, percutaneous coronary intervention; CABG, coronary artery bypass graft; ACE, angiotensin-converting enzyme; ARBs, angiotensin receptor blockers; LVEF, left-ventricle ejection fraction; RWMA, regional wall motion abnormalities; CFVR, coronary flow velocity reserve in the left anterior descending coronary artery; CACS, coronary artery calcium score. ^1^ Left main trunk with a stenosis ≥50% counted as two diseased vessels, and, if at least one of its main branches also showed stenosis >50%, this made for an overall count of three diseased vessels. Only the first event between cardiac death and nonfatal myocardial infarction was counted.

**Table 2:** Univariate and multivariate competing risk regression models for cardiac events (death or nonfatal myocardial infarction).

	Univariate Model		Multivariate Model			
	Crude HR (95% CI)	*p*-Value	Adjusted HR (95% CI)	*p*-Value	Adjusted HR (95% CI)	*p*-Value
Demographics and clinical risk factors						
Age, years	1.019 (0.988–1.051)	0.235				
Male gender	0.860 (0.479–1.542)	0.613				
Hypertension	0.824 (0.478–1.420)	0.818				
Hypercholesterolemia	0.731 (0.426–1.254)	0.255				
Smoke	1.249 (0.709–2.202)	0.441				
Diabetes	1.340 (0.735–2.445)	0.340				
Family history of CAD	1.293 (0.741–2.257)	0.365				
Known CAD ^1^	1.490 (0.746–3.319)	0.093				
Drugs						
Statins	2.050 (0.909–4.624)	0.084				
Beta-blockers	0.825 (0.522–1.304)	0.460				
Aspirin	1.631 (0.728–3.653)	0.235				
Echocardiography						
LVEF	0.954 (0.921–0.987)	0.007	1.004 (0.970–1.040)	0.815		
LVEF < 50	3.440 (1.917–6.173)	<0.001			1.577 (0.848–2.932)	0.150
Stress echocardiography						
Any reversible wall motion abnormality	7.189 (4.112–12.569)	<0.001	1.620 (0.722–3.635)	0.242	1.765 (0.946–3.291)	0.074
CFVR	0.034 (0.017–0.067)	<0.001	0.044 (0.014–0.142)	<0.001		
CVFR < 2	26.303 (8.413–64.552)	<0.001			13.890 (4.154–46.445)	<0.001
Coronary computed tomography angiography						
CACS (for 10-point increase)	1.004 (1.002–1.006)	<0.001	1.004 (1.002–1.006)	0.001		
CACS = 1–99	2.033 (0.457–9.037)	0.351			0.701 (0.192–2.557)	0.590
CACS = 100–399	5.912 (1.363–25.655)	0.018			1.193 (0.329–4.332)	0.788
CACS ≥ 400	7.208 (1.649–31.503)	<0.001	-	-	1.234 (0.319–4.768)	0.689
Number of vessels with coronary stenosis ≥ 50%	1.975 (1.591–2.453)	<0.001	0.980 (0.694–1.385)	0.910	1.070 (0.769–1.488)	0.689
Procedures after index exams						
Elective revascularization during follow-up	2.629 (1.525–4.531)	0.001	0.662 (0.350–1.253)	0.205	0.731 (0.392–1.365)	0.326

CAD, coronary artery disease; LVEF, left-ventricle ejection fraction; RWMA, regional wall motion abnormalities; CFVR, coronary flow velocity reserve in the left anterior descending coronary artery; CACS, coronary artery calcium score. Only one of the two parameters of wall motion assessment and one of the two of coronary calcium classifications included in univariate analysis were then tested in the multivariable model, due to the strict correlation between them. ^1^ Patients with previous myocardial infarction or/and elective revascularization.

## Data Availability

The datasets analyzed during the current study are available from the corresponding author on reasonable request.
